# P-55. Clinical Outcomes Associated with Once Daily IV Metronidazole Compared to More Frequent Administrations

**DOI:** 10.1093/ofid/ofaf695.284

**Published:** 2026-01-11

**Authors:** Rena A Nietfeld, Nick Bennett, Laura Aragon, Kensey Gosch, Sarah E Boyd

**Affiliations:** Saint Luke's Hospital of Kansas City, Department of Pharmacy, Leawood, KS; Saint Lukes Hospital of Kansas City, Kansas City, Missouri; Saint Luke's Health System, Kansas City, Missouri; Saint Lukes Hospital of Kansas City, Kansas City, Missouri; Saint Luke's Health System, Kansas City, Missouri

## Abstract

**Background:**

Metronidazole demonstrates concentration-dependent activity against anaerobes and is traditionally dosed intravenously (IV) at 500 mg every 8–12 hours. Its active metabolite maintains ∼60% of the parent drug’s activity and persists for 16–32 hours. Since 2009, Saint Luke’s Health System, standardized IV dosing to 1 g daily with few exceptions. Despite limited data, recent literature found 500 mg twice daily dosing was effective for anaerobic bacteremia. The purpose of this study was to compare metronidazole 1 gram IV once daily to metronidazole 500 mg IV at more frequent dosing schemes.

**Figure:**
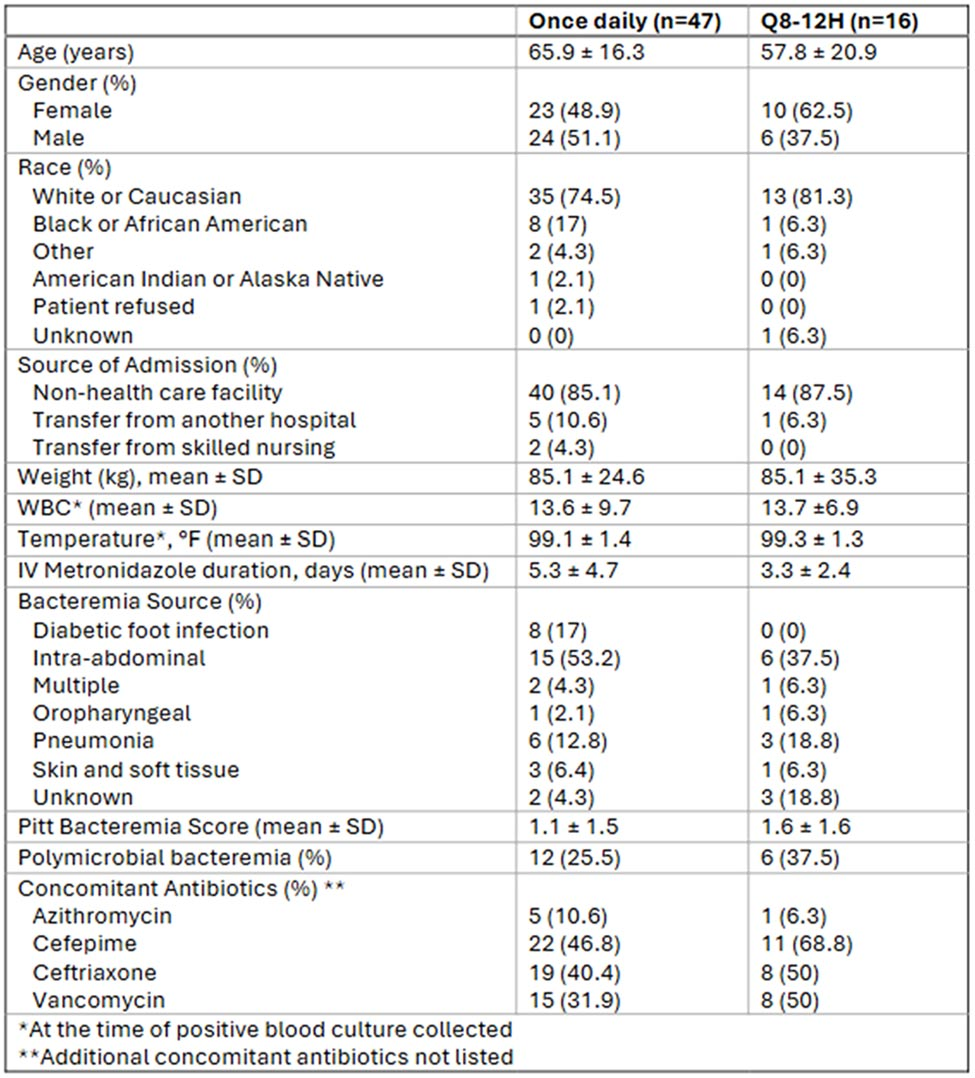
Baseline Characteristics

**Figure:**
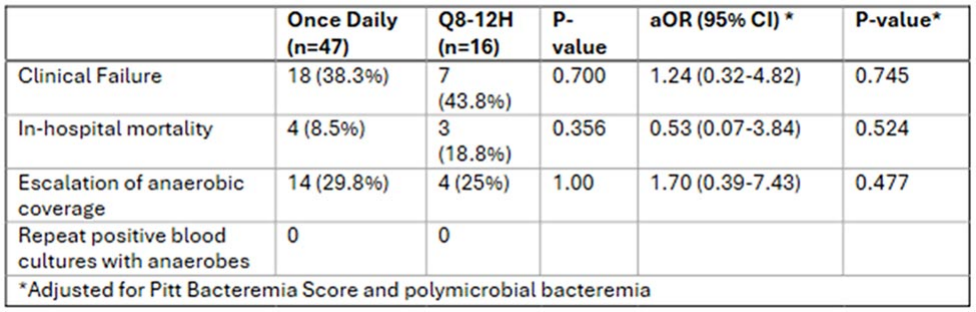
Primary Outcome

**Methods:**

This was a retrospective, multi-center, single health system study involving patients hospitalized between July 1, 2017, and December 31, 2024. Patients were included if they had culture confirmed anaerobic bacteremia and received metronidazole 1 gram IV once daily or 500 mg IV given q8-12 hours. Patients were excluded if they received concomitant therapy with agents that have activity against anaerobes, transitioned between metronidazole dosing approaches after 24 hours, or died within 24 hours of the positive blood culture collection time. The primary outcome was clinical failure, defined as a composite of all-cause inpatient mortality, change in anaerobic coverage, and repeat positive blood cultures with anaerobes. Secondary outcomes included ICU and hospital length of stay.

**Figure:**
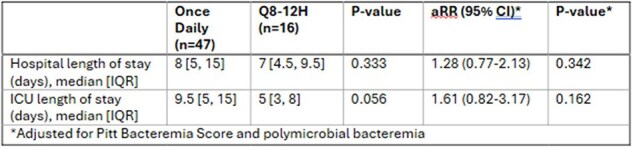
Secondary Outcomes

**Results:**

63 patients met inclusion (47 once daily, 16 q8–12h). Baseline characteristics were similar, although the once-daily group had lower Pitt Scores and fewer polymicrobial cases. No significant difference in clinical failure was observed (38.3% vs. 43.8%, p=0.700) including after adjusting for Pitt Bacteremia Score and polymicrobial bacteremia (aOR 1.24, 95% CI -1.13 to 1.57, p=0.745). ICU and hospital LOS were not different between groups (9.5 vs. 5 days, p=0.162; 8 vs. 7 days, p=0.342).

**Conclusion:**

In patients with anaerobic bacteremia, our data did not show a significant difference in clinical failure rate between metronidazole given 1 gram IV daily and those receiving more frequent dosing.

**Disclosures:**

All Authors: No reported disclosures

